# Pharmacogenetics of toxicity of 5-fluorouracil, doxorubicin and cyclophosphamide chemotherapy in breast cancer patients

**DOI:** 10.18632/oncotarget.24148

**Published:** 2018-01-10

**Authors:** Karolina Tecza, Jolanta Pamula-Pilat, Joanna Lanuszewska, Dorota Butkiewicz, Ewa Grzybowska

**Affiliations:** ^1^ Center for Translational Research and Molecular Biology of Cancer, Maria Sklodowska-Curie Memorial Cancer Center and Institute of Oncology, Gliwice, Poland

**Keywords:** breast cancer, FAC, chemotherapy, polymorphism, treatment toxicity

## Abstract

The differences in patients’ response to the same medication, toxicity included, are one of the major problems in breast cancer treatment. Chemotherapy toxicity makes a significant clinical problem due to decreased quality of life, prolongation of treatment and reinforcement of negative emotions associated with therapy.

In this study we evaluated the genetic and clinical risk factors of FAC chemotherapy-related toxicities in the group of 324 breast cancer patients. Selected genes and their polymorphisms were involved in FAC drugs transport (*ABCB1, ABCC2, ABCG2,SLC22A16*), metabolism (*ALDH3A1, CBR1, CYP1B1, CYP2C19, DPYD, GSTM1, GSTP1, GSTT1, MTHFR,TYMS*), DNA damage recognition, repair and cell cycle control (*ATM, ERCC1, ERCC2, TP53, XRCC1*).

The multifactorial risk models that combine genetic risk modifiers and clinical characteristics were constructed for 12 toxic symptoms. The majority of toxicities was dependent on the modifications in components of more than one pathway of FAC drugs, while the impact level of clinical factors was comparable to the genetic ones. For the carriers of multiple high risk factors the chance of developing given symptom was significantly elevated which proved the factor-dosage effect. We found the strongest associations between concurrent presence of clinical factors - overall and recurrent anemia, nephrotoxicity and early nausea and genetic polymorphisms in genes responsible for DNA repair, drugs metabolism and transport pathways. These results indicate the possibility of selection of the patients with expected high tolerance to FAC treatment and consequently with high chance of chemotherapy completion without the dose reduction, treatment delays and decline in the quality of life.

## INTRODUCTION

Breast cancer is heterogeneous disease with distinct molecular subtypes that require many therapeutic approaches. One of the major problems in breast cancer treatment are the differences in patients’ response to the same medication, including different side effects [1, 2]. Chemotherapy toxicity is a significant clinical problem due to decreased quality of life, prolongation of treatment and reinforcement of negative emotions associated with therapy. Many cancer patients decline future chemotherapy treatments and consider stopping chemotherapy altogether because of their fear of experiencing further toxicity and side effects [3].

The combination of fluorouracil, doxorubicin and cyclophosphamide, the FAC protocol, is a chemotherapy regimen commonly administered to breast cancer patients in Poland. The undeniable advantage of this therapy scheme is low cost of treatment, its proven efficacy and mostly acceptable toxicity. Usually FAC regimen is employed as adjuvant, neoadjuvant or palliative chemotherapy [4]. Its pharmacokinetics is very variable due to the complexity of the drugs’ action and the contribution of metabolizing, transporting and repairing proteins. At the same time, this complexity makes the genes and proteins of FAC pathways the ideal subjects of pharmacogenetics research. It is postulated that the variation, including single nucleotide polymorphisms (SNPs) in the phase I activations, phase II detoxification enzymes, and ABC membrane transporters, plays an important role in the efficacy and toxicities of chemotherapy [5]. The fact, that the weak genetic modifications like SNPs do have phenotypic impact on chemotherapeutics’ toxicity, could be explained by the narrow therapeutic index of such agents. It is thought that the toxicity depends almost solely on the genotype of the patients, while the treatment response is primarily determined by the tumor genome combined with patient’s intrinsic genetic characteristics [6]. The components of metabolic phase I are responsible for both activation and inactivation of FAC drugs. Doxorubicin is metabolized by the CBR (carbonyl reductase) enzymes to its active component. Similarly cyclophosphamide, the cell cycle nonspecific prodrug, requires activation by a number of different cytochrome P450 enzymes, mainly of CYP2C family [7]. Other important genes for cyclophosphamide pharmacokinetics belong to aldehyde dehydrogenase (ALDH) family, responsible for the shutting down of the active cyclophosphamide metabolites [8]. 5-fluorouracil also is the subject of deactivation driven by phase I enzymes, mainly by the *DPYD* gene product, the dihydropyrimidine dehydrogenase, which is the key rate-limiting enzyme for this drug [9]. The second, synthetic metabolism phase engages the glutathione transferases (GST), especially GSTA1, to drive the detoxification reactions of active cyclophosphamide metabolites. Also the disposition of active form of doxorubicin, the doxorubicinol, is influenced by the activity of GST enzymes [7]. Apart from metabolic pathways, the transport systems are also crucial for the treatment outcome, as both importers and exporters are responsible for the cellular drugs’ concentration. It is expected that any variation that affects transporter activity would be reflected in not only the response to treatment, but also in the development of drug-related toxicity. Doxorubicin entry to the cells is facilitated by the solute importer SLC22A16. Another member of solute carrier family, SLC22A7, has been correlated with the influx of 5-fluorouracil. In turn, the efflux of FAC drugs uses several ATP-binding cassette transporters (ABC), ABCB1, ABCC1, ABCC2, ABCG2. Alongside the genes and enzymes of metabolic pathways, the components of DNA repair systems, as well as the main drug targets and their modifiers, like 5-FU’s thymidylate synthase gene (TYMS) and MTHF, may influence adverse treatment reactions [7, 10–12].

Unfortunately, chemotherapy-induced toxicities are commonly affecting cancer patients with various intensity, and could be the reason for treatment delays and significantly lowered quality of life. Hematological and gastrointestinal toxicities are common in patients treated with cyclophosphamide and doxorubicin. Extremely high proliferative capacity of hematopoietic system makes it the collateral target for chemotherapeutic agents. Myelosuppression is the main dose-limiting toxicity in cancer treatment, yet myelotoxic regimes remain the current standard of breast cancer patients’ care. Chemotherapy-induced neutropenia and febrile neutropenia are frequent complications in cancer patients, because of the high susceptibility of neutrophil lineage to cytotoxic effects of cancer treatment. The drug-induced destruction of neutrophil precursors in bone marrow is the main cause of those symptoms. Decrease in neutrophil count is managed by the dose reduction and delays that decrease the dose intensity, whereas maintaining the dose is important for favorable response to treatment. Furthermore, patients with neutropenic events are more prone to subsequent infections [13–16]. Another frequent and serious myelotoxic symptom in breast cancer chemotherapy is anemia. This condition may emerge from the disease itself, but the effect of concomitant administration of cytotoxic drugs is also the cause of drop in the hemoglobin level. Anemia has deleterious effect on patients’ quality of life as well as on the treatment response. The suspected causes include blood loss, reduced or impaired erythrocytes production and high rate of red blood cells destruction or their reduced survival [17, 18]. Despite the improvements in cancer chemotherapy, kidney toxicity as the result of exposure to drugs remains one of the common complications. Apart from the liver, the kidneys are the main route of drugs’ excretion. These organs work under high blood pressure, in hypoxic environment and endure considerable concentrations of chemotherapeutic agents. There are several established clinical risk factors for nephrotoxicity in cancer patients, including renal drug handling, direct tumor impact on kidney structure, true or effective volume depletion and innate drug toxicity [19, 20]. Chemotherapy-induced nausea and vomiting (CINV) is a common severe side effect for cancer patients undergoing emetic chemotherapy [21, 22]. The complete pathophysiology of CINV is not known but gastrointestinal (GI) side effects associated with anticancer chemotherapy are traditionally thought to be attributable to mucosal damage [23]. Nausea is complex in nature and probably depending on more than one etiological factor [24]. Different pathways have been identified for acute and delayed CINV [25]. Also, nausea and vomiting can result in anorexia, decreased performance status, metabolic imbalance, wound dehiscence, esophageal tears and nutritional deficiency [26].

In our previous work we presented the multifactorial polymorphic genetic model of the response to FAC chemotherapy in breast cancer patients [27]. In the present study we focused on the analysis of the relations between the polymorphic variants in genes with known or potential role in the activity on FAC drugs and different symptoms of FAC-induced toxicity. We also looked into the possible impact of simultaneous presence of several variants on occurrence of side effects in order to establish multifactorial risk models. Single nucleotide polymorphisms were analyzed in genes encoding proteins involved in FAC drug transport (*ABCB1*, *ABCC2*, *ABCG2, SLC22A16*), metabolism (*CYP1B1*, *CYP2C19*, *GSTT1*, *GSTM1*, *GSTP1*, *TYMS*, *MTHFR*, *DPYD*), drug-induced damage repair (*ERCC1*, *ERCC2*, *XRCC1*) and in the regulation of DNA damage response and cell cycle control (*ATM*, *TP53*). Above listed set of genes and their variants was chosen to follow the route of doxorubicin, cyclophosphamide and 5-fluorouracil in cells, as well as the activity of cellular repair systems.

## RESULTS

Univariate analyses done for chosen genetic variants, clinical factors and symptoms of chemotherapy-related side effects revealed series of dependencies. Because of the great number of correlations, we present here the results of multivariate (Supplementary Table 1) and cumulative (Supplementary Table 1) analyses. The latter was conducted to study the effect of simultaneous presence of independent FAC toxicity risk factors. The aim of this study was to determine not solely the independent predictors, but also the risk factors of toxicity symptoms, for the genetic data the most frequent homozygote was not used as reference genotype in cases when polymorphic variant was the risk decreaser in univariate analysis. This approach enabled us to perform cumulative analyses only for risk enhancers and to build clear and interpretable cumulative models.

### Independent predictive factors of hematological toxicities

In multivariate analyses two or more genetic or clinical factors correlated with anemia (overall, recurrent, early), overall leukopenia and neutropenia (overall recurrent and severe recurrent), and was established as independent predictive factors (Supplementary Table 1).

Risk of FAC-related anemia of any grade was elevated by polymorphic allele C of p.Asn118= (rs11615) variant in *ERCC1* gene, common homozygote GG of *ABCC2* p.Val417Ile (rs2273697) polymorphism, presence of both *GSTT1* and *GSTM1* genes and triple-negativity of breast cancer. It should be emphasized that all three of the above listed genetic factors changed anemia risk at similar level (OR 4.01 – 4.58) and the impact of the only clinical component - triple-negativity- was definitely lower (OR 3.07; 95% CI 1.03–9.13). Also its statistical significance, while below the threshold of 0.05, was still the weakest one (*p* = 0.042) in this analysis (Supplementary Table 1). Accumulation of these factors was linked to growing risk of overall anemia (Supplementary Table 1). It should be pointed out that in the group lacking any of those high-risk factors (i.e. the 0’s group) the cases of anemia were not present. Therefore all the calculations were made for the group combined from 0’s and 1’s as the reference. The results showed, that the presence of two and more independent risk factors of anemia was responsible for over five-fold elevation of this symptom’s risk (OR 5.27; 95% CI 1.54–18.10; *p* = 0.008). Taken separately, when compared to reference group, the carriers of three unfavorable factors were at extremely high risk – OR 14.6 (95% CI 4.02–52.78; *p* = 0.00003). Furthermore, the risk of the group with all four factors was comparable (OR 12.0) to the 3’s, but the result was in the statistical range defined as trend (*p* = 0.055; 95% CI 0.92–156.0).

The recurrence of anemia in four or more cycles of FAC chemotherapy was connected to the presence of the rare allele G of variant p.Pro329Ala in *ALDH3A1* gene (rs2228100) and common homozygote CC of another ABC transporter gene polymorphism – *ABCB1* p.Ile1145= (rs1045642) (Supplementary Table 1). The results of cumulative analysis revealed that the carriers of both high-risk genotypes are over six times more likely to have anemia at 4 or more cycles of FAC treatment when compared to the group combined of the 1’s and 0’s. Similarly as in the overall anemia cumulative analysis, there were no cases of recurrent anemia in the group of non-carriers (Table 1).

**Table 1:** The association between accumulation of unfavorable genotypes and clinical factors and toxicity risk

				Toxicity			Toxicity risk OR (± 95% CI)	*p*
Symptom	Unfavorable genotypes and clinical factors		Number of unfavorable factors	absent *n* (%)	present *n* (%)	*p*		
ANEMIA grade 1-2	*ERCC1* p.Asn118= rs11615 *ABCC2* p.Val417Ile rs2273697 *GSTT1* and *GSTM1* deletions TNBC	TC/CC GG both genes present yes	0	21 (7.9)	--	<0.00001^*^	1 (ref.	
			1	87 (32.8)	3 (11.5)			
			2	112 (42.3)	5 (19.2)		1.61 (0.37-6.94)	0.523
			3	42 (15.8)	17 (65.4)		**14.6** (4.02-52.78)	**0.00003**
			4	3 (1.1)	1 (3.8)		12.0 (0.92-156.0)	0.055
			0-1	108 (40.7)	3 (11.5)	0.003^**^	1 (ref.)	
			2-4	157 (59.3)	23 (88.5)		**5.27** (1.54-18.10)	**0.008**
			0-3	262 (98.9)	25 (96.2)	0.314^**^	1 (ref.)	
			4	3 (1.1)	1 (3.8)		3.46 (0.35-35.18)	0.286
ANEMIA - RECURRENT grade 1-2	*ALDH3A1* p.Pro329Ala rs2228100 *ABCB1* p.Ile1145= rs1045642	CG/GG CC	0	101 (39.3)	--	0.005^*^	1 (ref.)	
			1	135 (52.5)	5 (62.5)			
			2	21 (8.2)	3 (37.5)		**6.74** (1.50-30.41)	**0.013**
ANEMIA – EARLY grade 1-2	*ABCG2* p.Gln141Lys rs2231142 *GSTT1* and *GSTM1* deletions	CA both genes present	0	137 (51.1)	2 (14.3)	0.0015^*^	1 (ref.)	
			1	85 (31.7)	11 (78.6)		**8.86** (1.90-41.30)	**0.005**
			2	46 (17.2)	1 (7.1)		1.49 (0.13-17.10)	0.747
			0	137 (51.1)	2 (14.3)	0.011^**^	1 (ref.)	
			1-2	131 (48.9)	12 (85.7)		**6.27** (1.37-28.76)	**0.018**
			0-1	222 (82.8)	13 (92.9)	0.478^**^	1 (ref.)	
			2	46 (17.2)	1 (7.1)		0.37 (0.05-2.93)	0.345
LEUCOPENIA grade 1-3	*ALDH3A1* p.Pro329Ala rs2228100 *CYP2C19* c.-806C>A rs12248560	CG/GG GG	0	34 (33.0)	21 (16.4)	0.0018^*^	1 (ref.)	
			1	48 (46.6)	58 (45.3)		**1.96** (1.00-3.82)	**0.048**
			2	21 (20.4)	49 (38.3)		**3.78** (1.78-8.04)	**0.0005**
			0	34 (33.0)	21 (16.4)	0.005^**^	1 (ref.)	
			1-2	69 (67.0)	107 (83.6)		**2.5**1 (1.34-4.69)	**0.004**
			0-1	82 (79.6)	79 (61.7)	0.004^**^	1 (ref.)	
			2	21 (20.4)	49 (38.3)		**2.42** (1.33-4.42)	**0.004**
NEUTROPENIA - SEVERE grade 3-4	*ABCC2* p.Ile1324= rs3740066 *TYMS* 28bp tandem repeat rs34743033 *DPYD* p.Ile543Val rs1801159	TT 3R/3R AA	0	32 (12.4)	5 (8.1)	0.0003^*^	1 (ref.)	
			1	127 (49.0)	18 (29.0)		0.91 (0.31-2.65)	0.858
			2	86 (33.2)	27 (43.5)		2.01 (0.71-5.72)	0.187
			3	14 (5.4)	12 (19.4)		**5.49** (1.59-19.0)	**0.006**
			0	32 (12.4)	5 (8.1)	0.506^**^	1 (ref.)	
			1-3	227 (87.6)	57 (91.9)		1.61 (0.60-4.32)	0.346
			0-2	245 (94.6)	50 (80.6)	0.001^**^	1 (ref.)	
			3	14 (5.4)	12 (19.4)		**4.20** (1.83-9.65)	**0.0007**
NEUTROPENIA – RECURRENT grade 1-4	*SLC22A16* p.Asn104= rs6907567 *CYP2C19* p.Pro227= rs4244285 *ERCC1* c.1510C>A rs3212986	AA GG AA	0	62 (27.1)	1 (4.4)	0.0001^*^	1 (ref.)	
			1	124 (54.2)	11 (47.8)		5.50 (0.69-44.13)	0.106
			2	42 (18.3)	9 (39.1)		**13.29** (1.59-111.32)	**0.016**
			3	1 (0.4)	2 (8.7)		**124.0** (5.21-2949.8)	**0.002**
			0	62 (27.1)	1 (4.4)	0.020^**^	1 (ref.)	
			1-3	167 (72.9)	22 (95.6)		**8.17** (1.07-62.49)	**0.042**
			0-2	228 (99.6)	21 (91.3)	0.023^**^	1 (ref.)	
			3	1 (0.4)	2 (8.7)		**21.7** (1.87-252.6)	**0.014**
NEPHROTOXICITY grade 1-2	*ERCC1* p.Asn118= rs11615 *GSTT1* and *GSTM1* deletions AGE	TC/CC both genes present post-menopausal (≥61yrs.)	0	47 (20.0)	--	<0.00001^*^	1 (ref.)	
			1	131 (47.3)	3 (21.4)			
			2	89 (32.1)	5 (35.7)		3.33 (0.78-14.36)	0.104
			3	10 (3.6)	6 (42.9)		**35.6** (7.67-165-20)	**<0.00001**
			0-1	178 (64.3)	3 (21.4)	0.003^**^	1 (ref.)	
			2-3	99 (35.7)	11 (78.6)		**6.59** (1.79-24.32)	**0.004**
			0-2	267 (96.4)	8 (57.1)	0.00002 ^**^	1 (ref.)	
			3	10 (3.6)	6 (42.9)		**20.02** (5.81-69.04)	**<0.00001**
GI – NAUSEA grade 1-3	*ATM* p.Asp1853Asn rs1801516 *CYP1B1* p. p.Leu432Val rs1056836 *GSTP1* p.Ile105Val rs1695	GA/AA CC AA/AG	0	11 (9.2)	5 (2.5)	0.0021^*^	1 (ref.)	
			1	66 (55.0)	88 (43.8)		2.93 (0.96-8.92)	0.056
			2	39 (32.5)	91 (45.3)		**5.13** (1.65-15.91)	**0.004**
			3	4 (3.3)	17 (8.4)		**9.35** (1.94-45.04)	**0.004**
			0	11 (9.2)	5 (2.5)	0.014^**^	1 (ref.)	
			1-3	109 (90.8)	196 (97.5)		**3.96** (1.33-11.73)	**0.013**
			0-2	116 (96.7)	184 (91.6)	0.101^**^	1 (ref.)	
			3	4 (3.3)	17 (8.4)		2.68 (0.88-8.20)	0.082
GI – NAUSEA EARLY grade 1-3	*ABCC2* p.Ile1324= rs3740066 *SLC22A16* p.His49Arg rs714368 N (nodes)	CT/TT AG/GG 1	0	13 (9.2)	--	0.00003^*^	1 (ref.)	
			1	63 (44.4)	45 (33.3)			
			2	54 (38.0)	61 (45.2)		**1.91** (1.13-3.22)	**0.015**
			3	12 (8.4)	29 (21.5)		**4.08** (1.88-8.84)	**0.0003**
			0-1	76 (53.5)	45 (33.3)	0.001^**^	1 (ref.)	
			2-3	66 (46.5)	90 (66.7)		**2.30** (1.41-3.75)	**0.0008**
			0-2	130 (91.6)	106 (78.5)	0.004^**^	1 (ref.)	
			3	12 (8.4)	29 (21.5)		**2.96** (1.43-6.10)	**0.003**
GI – NAUSEA EARLY SEVERE grade 3	*XRCC1* p.Arg399Glu rs25487 AGE	GA/AA pre-menopausal (≤39 yrs.)	0	121 (40.2)	6 (28.6)	<0.00001^*^	1 (ref.)	
			1	171 (56.8)	9 (42.8)		1.06 (0.36-3.06)	0.912
			2	9 (3.0)	6 (28.6)		**13.4** (3.56-50.85)	**0.0001**
			0	121 (40.2)	6 (28.6)	0.360^**^	1 (ref.)	
			1-2	180 (59.8)	15 (71.4)		1.68 (0.63-4.47)	0.296
			0-1	292 (97.0)	15 (71.4)	0.0001^**^	1 (ref.)	
			2	9 (3.0)	6 (28.6)		**13.0** (4.07-41.4)	**0.00001**
GI – VOMITING RECURRENT grade 1-3	*CBR1* p.Ala209= rs20572 *TP53* p.Arg72Pro rs1042522	CC GG	0	229 (92.3)	1 (20.0)	0.0003^**^	1 (ref.)	
			1	19 (7.7)	4 (80.0)		**48.2** (5.07-458.1)	**0.0007**
			2	--	--		--	--
GI – VOMITING EARLY SEVERE grade 3	*ATM* p.Asp1853Asn rs1801516 AGE	AA pre-menopausal (≤39 yrs.)	0	282 (91.0)	6 (50.0)	0.00001^*^	1 (ref.)	
			1	26 (8.4)	5 (41.7)		**9.04** (2.57-31.79)	**0.0006**
			2	2 (0.6)	1 (8.3)		**23.5** (1.85-299.04)	**0.014**
			0	282 (91.0)	6 (50.0)	0.0005^**^	1 (ref.)	
			1-2	28 (9.0)	6 (50.0)		**10.07** (3.03-33.47)	**0.0002**
			0-1	308 (99.4)	11 (91.7)	0.108^**^	1 (ref.)	
			2	2 (0.6)	1 (8.3)		**14.0** (1.17-167.84)	**0.037**

^*^ Pearson χ^2^ test; ^**^Fisher two-way exact test; OR- odds ratio; 95% CI- confidence interval; bolded numbers indicate results with *p <* 0.05; TNBC- triple negative breast cancer.

The variation in another of ABC transporters gene, *ABCG2*, was also responsible for early anemia, seen in two first courses of treatment. Heterozygote CA of p.Gln141Lys (rs2231142) variant increased risk of early anemia nearly 4 times (OR 3.72; 95% CI 1.30–10.66; *p* = 0.014), together with the presence of both *GSTT1* and *GSTM1* genes (OR 3.13; 95% CI 1.04–9.40; *p* = 0.041). In our group the rare homozygote AA of *ABCG2* p.Gln141Lys variant was not present (Supplementary Table 1). Taken together, the presence of at least one high-risk factor, when compared to the non-carriers, was responsible for elevation of the early anemia risk to OR 6.27 (95% CI 1.37–28.76; *p* = 0.018). The impact of the group carrying CA heterozygote of *ABCG2* p.Gln141Lys variant and both *GSTT1*/*GSTM1* genes was not statistically significant (Table 1).

The multivariate analysis of leukopenia of any grade during the FAC treatment revealed two genetic independent risk factors. Both of them changed the risk about twofold – OR 1.93 (95% CI 1.13–3.31; *p* = 0.016) by variant allele G of p.Pro329Ala in *ALDH3A1* gene (rs2228100) and OR 1.96 (95% CI 1.13–3.36; *p* = 0.014) by *CYP2C19* c.-806C>A (rs12248560) common homozygote CC (Supplementary Table 1).The results of cumulative analysis done for overall leukopenia revealed strong progressive rise of its risk with growing number of unfavorable factors (Table 1). The carriers of one of those genotypes were at nearly twofold risk (OR 1.96; 95% CI 1.00–3.82; *p* = 0.048), but the concomitance of both of them resulted in further doubling the risk to the OR 3.78 (95% CI 1.78–8.04; *p* = 0.0005). The 2’s group analyzed in comparison to non-carriers and the 1’s combined, was endangered by the FAC-related leukopenia at the level of OR 2.42 (95% CI 1.33–4.42; *p* = 0.004). Nearly identical result was obtained for the groups of carriers of at least one factor (the 1’s and the 2’s) in relation to the group without any of them (OR 2.51; 95% CI 1.34–4.69; *p* = 0.004).

Severe neutropenia of grades 3 and 4 had three genetic factors as independent predictors that changed the risk in a similar way (Supplementary Table 1). The strongest one was the 3R3R variant of *TYMS* 28bp tandem repeat (rs34743033), which over-doubled the risk (OR 2.16; 95% CI 1.20–3.88; *p* = 0.010). The second predictor was another studied polymorphism of *ABCC2*– p.Ile1324= (rs3740066). Its variant homozygote TT elevated risk of severe neutropenia to OR 1.92 (95% CI 1.09–3.41; *p* = 0.024). The last factor in this analysis, the presence of common homozygote AA of *DPYD* p.Ile543Val (rs1801159), had the impact on the symptom’s risk on the borderline significance (OR 1.89; 95% CI 0.97–3.67; *p* = 0.059). Nonetheless, this polymorphism was left in the multivariate model because the overall significance of the model with the *DPYD* variant was stronger than the model without this factor (*p* = 0.002 vs. 0.005; data not shown). In cumulative analyses, the carriers of all three independent factors of severe neutropenia had over five times higher risk of this symptom than the non-carriers (OR 5.49; 95% CI 1.59–19.0); *p* = 0.006) (Table 1). This effect, even statistically stronger, was present also in comparison of the 3’s with the group combined from 0’s, 1’s and 2’s (OR 4.20; 95% CI 1.83–9.65; *p* = 0.0007).

Apart from the ABC family, another transporter gene established as independent predictive factor for hematological toxicities - recurrent neutropenia, was *SLC22A16* (Supplementary Table 1). The p.Asn104= (rs6907567) common homozygote AA was responsible for over three-fold increase in the risk of this symptom (OR 3.15; 95% CI 1.00–9.92; *p* = 0.049), together with polymorphic allele A of the *CYP2C19* p.Pro227= variant (rs4244285; OR 2.85; 95% CI 1.15–7.05; *p* = 0.023). However, the strongest factor for the recurrent neutropenia was the rare homozygote AA of the *ERCC1* c.1510C>A polymorphism (rs3212986), responsible for the over 5-times higher risk when compared to genotypes with common allele C (OR 5.45; 95% CI 1.62–18.29; *p* = 0.006). Simultaneous presence of two of these three independent predictive factors was responsible for a very high risk of recurrent neutropenia in four or more cycles of treatment (OR 13.29; 95% CI 1.59–111.32; *p* = 0,016) (Table 1). The risk for the 3’s group, when compared to non-carriers, was enormous (OR 124.0; 95% CI 5.21–2949.8; *p* = 0.002). The 3’s were also at very high risk of recurrent neutropenia in relation to the reference group combined from 0’s, 1’s and 2’s (OR 21.7; 95% CI 1.87–252.6; *p* = 0.014). It should be noted however, that these results – while strongly significant – are unreliable due to extremely wide confidence interval. Nonetheless, cumulative analysis proved that the risk of recurrent neutropenia for the carriers of any of the established independent risk factors is greatly increased and reaches OR 8.17 (95% CI 1.07–62.49; *p* = 0.042).

### Independent predictive factors of kidney damage – nephrotoxicity

In our group elevated creatinine level as the equivalent of nephrotoxicity of grades 1-2 had two genetic and one clinical independent predictors (Supplementary Table 1). In this case, opposite from the anemia analysis, the stronger influence on toxicity risk had the clinical factor – age at the time of diagnosis. Women in the post-menopausal age (≥61 yrs) had over 7-fold higher risk of nephrotoxicity of FAC treatment than the younger patients (OR 7.42; 95% CI 2.27–24.28; *p* = 0.0009). From the genetic perspective, contributors to kidney damage risk were the polymorphic allele C of *ERCC1* variant p.Asn118= (rs11615) and the presence of both *GSTT1* and *GSTM1* genes. Those factors influenced the symptoms risk in a similar way – OR 4.73 (95% CI 0.99–22.57; *p* = 0.051) and OR 4.81 (95% CI 1.37–16.83; *p* = 0.014), respectively. It should be noted that like in the analysis of the risk of severe neutropenia, the variant with borderline significance (i.e. *ERCC1*p.Asn118=) was left in the model because the significance of this calculation was stronger than when this factor was removed (*p* = 0.0009 vs. 0.0061; data not shown). The cumulative analysis revealed that when all three factors were present at the same time, the risk of nephrotoxicity was drastically increased (OR 35.6; 95% CI 7.67–165.20; *p <* 0.00001) (Table 1). Due to the lack of cases with elevated creatinine level among the non-carriers, in this analysis the 0’s and the 1’s in the reference group were combined. Similarly, strongly significant high risk described the 3’s when compared to the other groups, i.e. 0’s-2’s (OR 20.02; 95% CI 5.81–69.04; *p <* 0.00001). Generally the results showed, that the presence of at least 2 unfavorable factors was responsible for over six-fold increase in the risk of negative effect of FAC chemotherapy on kidneys (OR 6.59; 95% CI 1.79–24.32; *p* = 0.004). It is crucial to point out that in our group the occurrence of FAC nephrotoxicity was not related to dehydration due to nausea and vomiting (respectively *p* = 1.000 and *p* = 0.219; data not shown).

### Independent predictive factors of gastrointestinal toxicity

The multivariate analyses done for toxic symptoms from the digestive tract revealed two or more independent factors for overall, early, early severe nausea and for recurrent and early severe vomiting (Supplementary Table 1).

Three genetic predictors of overall nausea were responsible for the doubling of the symptom risk. The most frequent homozygote CC of the *CYP1B1* p.Leu432Val polymorphism had the strongest statistical significance in this analysis (OR 1.91; 95% CI 1.15–3.16; *p* = 0.012). The two other polymorphic variants - rare allele A of *ATM* p.Asp1853Asn (rs1801516) and common allele A of *GSTP1* p.Ile105Val (rs1695), influenced nausea risk with significance close to the threshold. For these polymorphisms the nausea risk was established as OR 1.79 (95% CI 1.01–3.19; *p* = 0.046) and OR 2.20 (95% CI 1.00–4.82; *p* = 0.049), respectively (Supplementary Table 1). The risk of this symptom was also progressively increasing with the growing number of high-risk genotypes (Table 1). The result of the calculations opposing the non-carriers with the patients with one overall nausea predictor was at the borderline significance (OR 2.93; 95% CI 0.96–8.92; *p* = 0.056), but the presence of two unfavorable genotypes resulted in the significant risk of OR 5.13 (95% CI 1.65–15.91; *p* = 0.004). The carriers of all three variants had the risk over nine times higher (OR 9.35; 95% CI 1.94–45.04; *p* = 0.004). When we confronted all the carrier groups (1’s - 3’s) with the 0’s, the chances of developing overall nausea were at the level of OR 3.96 (95% CI 1.33–11.73; *p* = 0.013).

The analysis of nausea in the first two cycles of treatment revealed one clinical and two genetic predictors (Supplementary Table 1). The clinical one was the presence of regional lymph node metastases (N1 from TNM staging) and was connected with early nausea risk at the level of OR 1.89 (95% CI 1.14–3.16; *p* = 0.014). Similar impact had the polymorphic allele G of *SLC22A16* transporter gene variant p.His49Arg (rs714368; OR 1.78; 95% CI 1.07–2.95; *p* = 0.025). However, the variation in another transporter gene, *ABCC2* p.Ile1324= (rs3740066) was the strongest independent factor in this analysis. The presence of its rare allele T brought over fourfold strongly significant risk elevation – OR 4.49 (95% CI 1.85–10.93; *p* = 0.0009). Similarly as with the overall nausea cumulative analysis, the concomitant presence of two and three high-risk independent factors was responsible for growing early nausea risk - from OR 1.91 (95% CI 1.13–3.22; *p* = 0.015) for the 2’s, to OR 4.08 (95% CI 1.88–8.84; *p* = 0.0003) for the 3’s (Table 1). Like in the few previous cumulative analyses, there were no cases of studied symptom in the group of non-carriers, and the reference group was combined from 0’s and 1’s. For the presence of at least two unfavorable factors the chances of nausea in the first two FAC cycles reached the OR 2.30 (95% CI 1.41–3.37; *p* = 0.0008). Comparable risk, yet statistically weaker, was linked to the 3’s alone in confrontation with the other groups (OR 2.96; 95% CI 1.43–6.10; *p* = 0.003).

Pre-menopausal age (≤39 yrs.) proved to be a strong risk factor for severe nausea in the two first cycles of FAC treatment (Supplementary Table 1). When compared to the older patients, the risk of said symptom reached OR 5.61 (95% CI 1.92–16.41; *p* = 0.002). Genetic independent factor that accompanied the patients’ age was the rare allele A of p.Arg399Glu (rs25487) in the *XRCC1* gene. When those two factors were present together, the cumulative risk reached the OR 13.4 (95% CI 3.56–50.85; *p* = 0.0001) when compared to non-carriers, and OR 13.0 (95% CI 4.07–41.4; *p* = 0.00001) with the other groups as the reference (Table 1).

Recurrent vomiting in our group was seen only in 5 patients, therefore the results of multivariate analysis had very wide confidence intervals and enormous ORs (Supplementary Table 1). Nonetheless, rare homozygotes of two polymorphisms were the independent predictors of recurrent vomiting – *CBR1* p.Ala209= (rs20572; OR 57.25; 2.98–1101.48; *p* = 0.007) and *TP53* p.Arg72Pro (rs1042522; OR 45.8; 4.44–472.56; *p* = 0.001). In our group there were no carriers of those two genotypes, and in the cumulative analysis the only calculation possible was for the 1’s group (Table 1). Unsurprisingly, the risk of recurrent vomiting for this group did not differ greatly from the results of multivariate analyses (OR 48.2; 95% CI 5.07–458.1; *p* = 0.0007).

For early severe vomiting, similarly as in nausea analysis in the same setting, patients’ young age (i.e. pre-menopausal, ≤39 yrs.) was strong predictive factor (Supplementary Table 1). These patients had the risk at the level of OR 5.06 (95% CI 1.29–19.78; *p* = 0.019). In this analysis however, the impact of genetic factor was even stronger, the rare homozygote AA of *ATM* p.Asp1853Asn (rs1801516) resulted in over nine-fold increase in early severe vomiting risk (OR 9.31; 95% CI 1.94–45.25; *p* = 0.005). When these two factors were present together, they contributed to early severe vomiting risk at the level of OR 23.5 (95% CI 1.85–299.04; *p* = 0.014) when compared to the 0’s, and of OR 14.0 (95% CI 1.17–167.84; *p* = 0.037) with the combined group as the reference (Table 1). The cumulative analysis revealed, that the presence of one or at least one high-risk factor was responsible for about ten-fold increase in the risk of studied symptom - OR 9.04 (95% CI 2.57–31.79; *p* = 0.0006) and OR 10.07 (95% CI 3.03–33.47; *p* = 0.002), respectively. It should be noted however, that because of the uneven distribution of cases in the cumulative groups, the confidence intervals were very wide and any conclusions must be very cautious.

## DISCUSSION

In the present work we evaluated the genetic and clinical risk factors of FAC chemotherapy-related toxicities in breast cancer patients, combined in three major groups - nephrotoxicity, myelotoxicity and gastrointestinal. Genes and their variants chosen for these analyses are responsible for cellular mechanisms of DNA damage detection, repair or cell cycle control, drugs’ metabolism, targets and transport. Additionally, the clinical characteristics of patients was also included to complete the picture.

Independent genetic factors responsible for the elevated risk of majority of analyzed toxicity symptoms established in this work, belong to at least two functional groups, which emphasizes the complex nature of each toxic symptom, as well as pleiotropic activity of genes and gene variants.

### Polymorphisms in DNA damage repair and cycle control genes

In this study polymorphic variants in *ERCC1*, *ATM*, *TP53* and *XRCC1* genes influenced the risk of FAC toxicity. ERCC1 protein is the key enzyme of the NER and ICL repair machinery. It is therefore intensively analyzed, both its expression and common variants, as the potential marker of cancer risk, chemotherapy responsiveness and side effects [28, 29]. ERCC1 is also crucial for the repair of cyclophosphamide-induced DNA damage and cells, both normal and cancerous, and low-activity mutants may be hypersensitive to DNA crosslinking agents [30]. Two *ERCC1* polymorphisms selected for this study, p.Asn118= (rs11615) in exon 4 and c.1510C>A (rs3212986) in 3’UTR are extensively studied in many cancers and chemotherapeutic regimes because of their negative influence on the stability and level of *ERCC1* mRNA and on the protein expression [31]. Silent variant p.Asn118= is associated with lower protein’s expression, transcript stability and protein levels [32]. It is believed that worse capacity of DNA repair in cancer patients is reflected in better response to genotoxic treatment due to damage accumulation in cancer cells. Unfortunately, as the chemotherapy impact on the body is systemic, the normal cells with innate low DNA repair also suffer from the treatment [33]. Our results support this assumption, as the rare C allele was connected with higher risk of nephrotoxicity. Concordant results come from the study by Khrunin [34], where heterozygotes were at increased risk of nephrotoxicity in ovarian cancer patients receiving cisplatin/gemcitabine chemotherapy. *ERCC1* polymorphisms were also responsible for elevated risk of FAC-induced overall anemia (p.Asn118=) and recurrent neutropenia (c.1510C>A). Similar observations regarding the p.Asn118= were made by the group of Lambrechts [35] in ovarian cancer patients receiving paclitaxel and carboplatin. They noted that the risk of anemia grade 3–4 was increased in the presence of this polymorphism. Concordant reports came from the studies of the gastroesophageal adenocarcinoma treated with oxaliplatin, where both variants were deemed the predictors of grade 3–4 anemia, leukopenia and neutropenia [36]. These results indicate, that active ERCC1 is crucial for the hematopoietic function of bone marrow. The support for this assumption came from the experiments on the *Ercc1^−/−^* mice. The group of Prasher [37] first demonstrated that basal hematopoiesis and reserve capacity were severely reduced in *Ercc1*-defficient mice. The mice had also multilineage cytopenia and bone marrow steatosis, the characteristic features of age-related functional decline of hematopoietic system. Similar study on *Ercc1^−/−^* mice confirmed the loss of hematopoietic reserves, reduced competitiveness of cells and failure of remaining progenitors of *in vitro* proliferation [38]. The authors also stated that hematopoietic defects are reminiscent of those in Fanconi Anemia patients. Taken together, we can hypothesize that high risk of hematologic toxicities seen in our group in carriers of variant alleles of *ERCC1* p.Asn118= and c.1510C>A can arise from the SNPs-related reduction of *ERCC1* expression. The impact of these polymorphic variants on the hematopoietic system must be of course much milder than that of complete gene knockout in experimental setting, but nonetheless may be revealed after systemic exposure to highly cytotoxic agents.

*ATM*, *TP53* and *XRCC1* polymorphic variants were the independent factors of gastrointestinal toxicities - nausea, recurrent vomiting and of severe early nausea and vomiting. Most researches focus on the polymorphisms analysis of these genes in terms of radiosensitivity and radiation toxicity. *ATM* and *TP53* genes participate in DNA damage detection and repair, and also can halt the cell cycle or induce cellular apoptosis. Furthermore, there is a strong connection between those genes, as the protein p53 is phosphorylated by ATM kinase shortly after DNA damage, resulting in enhanced stability and activity of p53 [39–42]. XRCC1 protein plays a key role in the BER pathway that is involved in repair of DNA single-strand breaks after exposure to reactive oxygen species (ROS), ionizing radiation (IR) or alkylating agents, but also takes part in repairing the double-strand breaks [43]. Unfortunately, the literature data do not explain comprehensively the relationship between CINV and genetic background, including the DNA damage repair-related one. Probably, genes of this functional group might be involved in inflammation and thus toxicity.

The *TP53* p.Arg72Pro (rs1042522) polymorphism is located in the proline-rich region of p53 and the substitution of arginine with proline can directly affect the structure of the putative SH3-binding domain. The Arg72 protein is more efficient in apoptosis induction, whereas the Pro72 form induces more G1 arrest and is better at activating p53-dependent DNA repair [34, 43]. In our study homozygote variant CC (Pro/Pro) predisposed to recurrent vomiting grade 1–3. Similarly in the work of Khrunin *et al*. severe neutropenia after cisplatin-based chemotherapy in ovarian cancer was more frequent among patients with a *TP53* variant homozygote CC (Pro/Pro) [34]. Also, the correlation between the*TP53* p.Arg72Pro polymorphism and acute skin toxicity after radiotherapy in breast cancer patients was observed by Tan *et al*. [44].

The variant p.Asp1853Asn (rs1801516) in *ATM* gene in our study changed the risk of two symptoms of gastrointestinal toxicity – nausea grade 1–3 and early severe vomiting. Andeasser *et al*. performed a meta-analysis on the relationship between *ATM* rs1801516 SNP and radiotherapy side effects in breast and prostate cancer patients. They observed strong impact of studied polymorphism on treatment-induced toxicity, mainly in skin and mucosal membranes. The authors hypothesized, that the change of acidic aspartic acid in position 1853 to the polar asparagine formed the tendency towards cell cycle arrest or apoptosis, which could possibly lead to increased acute toxicity, but at the same time could be protective against radiation-induced malignancy. However, because of non-conclusive data about actual functional impact of this polymorphism on ATM kinase activity, variant p.Asp1853Asn could be as well as tagging SNP of another allele [45].

Early severe nausea had only one genetic predictor in our group, the rare allele A of polymorphism p.Arg399Glu (rs25487) in *XRCC1* gene. Similarly like in the above described variants, there is no conclusion about its exact function. There are experimental data showing that the variant genotype results in less efficient repair of DNA damage induced by various agents including chemicals, light, and irradiation [46]. Also, it has been presented that *XRCC1* 399Glu variant allele (A) had higher levels of DNA adducts [47] and tobacco-related DNA damage [48, 49]. Opposite to that, the group of Yarosh *et al*. described the sensitization of cytotoxic agents in the presence of GG common homozygote (Arg/Arg) and developing resistance for genotypes with variant allele A (Glu) [50]. In terms of chemotherapy-related toxicity, the study of Wang *et al*. supports our results. They showed that in the presence of p.Arg399Glu allele A the lung cancer patients treated with cisplatin-based chemotherapy had significantly increased risk of severe gastrointestinal toxicity [51]. Similarly, Peng and colleagues demonstrated that nonsmall-cell lung cancer patients treated with platinum-based chemotherapy and carrying at least one variant allele of *XRCC1* p.Arg399Glu had increased risk of hematological toxicity grade 3–4 [52]. Also, Liu *et al* identified the A allele as significantly correlated with grade 3 or 4 hematological toxicity in gastric patients receiving oxaliplatin-based adjuvant chemotherapy, which may have been due to the less proficient DNA repair activity [53].

### Polymorphisms in genes belonging to metabolic pathways of FAC drugs

FAC-induced toxicity in our study was found to be connected with variability in nine genes belonging to metabolic pathways of 5-FU, doxorubicin and cyclophosphamide. The variants were located in the phase I (*CYP2C19*, *CYP1B1*) and phase II (*GSTT1*, *GSTM1*, *GSTP1*) enzymes genes, in drugs metabolizers (*CBR1*, *ALDH3A1*, *DPYD*) and in 5-FU cellular target (*TYMS*). The polymorphisms in genes of cytochrome P450 influenced the hematological and gastrointestinal toxicity. Both variants in *CYP2C19* gene analyzed in this work, c.-806C>A (rs12248560; *17) and p.Pro227= (rs4244285; *2), correlated with leukopenia and recurrent neutropenia, respectively. There are some evidences, that those variants have impact on the ability of CYP2C19 to metabolize its substrates, including cyclophosphamide. The CYP2C19*17 promoter SNP causes the increase in transcriptional activity and forms the ultrarapid metabolizer phenotype. Conversely, the CYP2C19*2 variant has aberrant mRNA splicing, resulting in the absence of hepatic enzyme and poor metabolizer phenotype [54]. Polymorphisms’ functions give some support to our results, where the high risk genotypes were the ones linked to weaker enzyme activity, meaning the wild type homozygote of *17 and genotypes with variant allele A of *2. It is assumed that slower metabolism of cyclophosphamide results in toxic drug accumulation in normal tissues. Unfortunately, the results obtained by different research groups are not conclusive. On the one hand it has been shown, that the carriers of CYP2C19*2 and *3 have the reduced elimination rate of another CYP2C19 substrate, the sulfonamide anticancer drug indisulam. On the other these patients were also at high risk of severe neutropenia [55]. Negative conclusions were made by the group of Tulsyan *et al*. [56] in the group of breast cancer patients receiving cyclophosphamide, where there was no association with anemia and leukopenia. Similar lack of such correlations in cancer patients was reported by Bray *et al*. and Ekhart *et al*. [10, 57].

In our group the occurrence of nausea grade 1–3 was correlated with common CC homozygote of polymorphism p.Leu432Val (rs1056836; allele CYP1B1*3) in metabolic phase 1 gene *CYP1B1*. CYP1B1 is involved in the hydroxylation of estrogens, as well as in transformation of many compounds, including taxanes and doxorubicin. Variant p.Leu432Val is located in exon 3, which encodes the heme-binding domain. Functionally, it is associated with altered *CYP1B1*mRNA expression, as has been shown in the work of Gu *et al*. [58]. The authors showed that p.Leu432Val CC homozygote was significantly associated with higher expression levels of *CYP1B1* mRNA in biochemical recurrence in prostate cancer patients. There are also evidences of correlation of this variant with taxanes’ toxicity. The group of Boso reported that CYP1B1*3 is associated with stomatitis and mucositis in paclitaxel-treated breast cancer patients [59]. Also, Marsh *et al*. showed that CYP1B1 polymorphism in ovarian cancer patients treated with docetaxel was connected with grade ≥3 overall GI toxicity [60]. For the anthracyclines, *CYP1B1* seems to have an important role in doxorubicin cardiac toxicity. Maayah and colleagues demonstrated attenuation of cardiotoxicity after CYP1B1 inhibition [61]. Although in our group the cardiotoxicity of FAC chemotherapy was not analyzed, above cited results support the connection between *CYP1B1* expression-changing SNPs and chemotherapy outcome.

In our study homozygous deletion of *GSTT1* and *GSTM1* genes correlated with nephrotoxicity and anemia analyzed in two settings but, surprisingly, the independent risk factor was not the lack of those genes but their presence. The absence of enzymes due to *GSTT1* and *GSTM1* deletions is associated with reduced metabolism and detoxification rate of chemotherapeutic agents. It is usually expected then, that the absence of GST enzymes will result in increased drug concentration and better therapeutic effect combined with higher risk of adverse reaction [62, 63]. Similar situation to the one described here, where the very presence of GST genes was linked to treatment toxicity, was reported for the myelosuppression and neuropathy in breast and ovarian cancer. The former work showed that for the breast cancer patients the myelosuppression caused by CAF/CEF chemotherapeutic regimes was more frequent in patients with present genotype, although this observation was not statistically significant [64]. The latter study presented comparable, yet strongly significant, dependence. The authors evaluated the risk of side effects of cisplatin and cyclophosphamide treatment in ovarian cancer patients. They showed that the homozygous deletion of *GSTM1* gene decreased the risk of thrombocytopenia, anemia and neuropathy when compared to such risk in carriers of functional *GSTM1* variant [34]. The explanation of these and our observations may lie in the complex role of GST enzymes in the cell. They are regarded as parts of the detoxification system which, by conjugation with glutathione (GSH), neutralizes xenobiotics, including chemotherapeutic agents, therefore their expression is high in detox organs like liver and kidneys. Glutathione-S-transferases have also other, nonenzymatic roles, as they participate in protein-protein interactions with members of MAP kinases. Because of the handling of GSH, the GST enzymes are indirectly responsible for the intracellular redox balance and proteins’ thiol status. Moreover, GSH-dependent enzymes are important players in antioxidant defense, because the glutathione donates hydrogen to damaged molecules, reduces H_2_O_2_ and lipid peroxides [65, 66]. In the light of our results it could be assumed that fully functioning GSTT1 and GSTM1 enzymes are causing the decrease of GSH intracellular level, which in turn is responsible for tissue damage. The experiments on mouse model showed that GSH has protective effect against 5-FU-induced myelotoxicity [67]. Such effect was also reported by the group of Zhang, who observed that cisplatin cytotoxicity is regulated by the concentration of GSH in HepG2 cells, and the administration of GSH-lowering agent is reflected by the growing rate of cells’ death [68]. Similar results were obtained by Szwed *et al*. who showed, that the treatment of cancer cells with doxorubicin resulted in dramatic glutathione depletion [66]. They hypothesized, that this phenomenon could be the result of active detox system involving the GST enzymes, but also of the oxidative properties of the drug itself, as the formation of reactive oxygen species (ROS) is the main mechanism of doxorubicin cytotoxicity. Interestingly, other experiments on mice also revealed that the cyclophosphamide dosing is the reason of significant depletion of GSH in bone marrow, liver and, at high concentration, in the blood [69]. Physiologically, the red blood cells are in a high level of oxidative stress as they transport large amounts of oxygen, and they are in need of a significant amount of antioxidants. Furthermore, the mature cells do not have the nucleus, therefore they rely, among others, on the enzymes of the glutathione system [70]. In the condition of oxidative stress, the erythrocytes have shortened life, could undergo hemolysis with the possible effect of anemia [71].

The *GSTP1* p.Ile105Val (rs1695) wild homozygote AA was the independent nausea grade 1-3 predictor in our study. This polymorphism has been extensively analyzed in many conditions and in relation to several drugs, but the results are inconclusive. Zhong and others observed the significantly increased risk of myelosuppression and gastrointestinal toxicity in the presence of Val/Val (GG) genotype in patients with lupus erythematosus treated with cyclophosphamide. The authors suggested that the *GSTP1*polymorphism at codon 105 resulted in reduced enzyme activity towards cyclophosphamide and its toxic metabolites. These metabolites would accumulate in the body, leading to increased short term adverse reactions [72]. Opposite findings come from the work of Liu and colleagues. In this study on the gastric cancer patients receiving oxaliplatin-based treatment, patients with GSTP1 p.Ile105Val A allele had a higher incidence of grade 3-4 cumulative neuropathy, gastrointestinal toxicity and hematological toxicity [53]. Our results are in accordance with the report of Liu *et al*. It could be hypothesized, that the presence of allele A of *GSTP1* p.Ile105Val is connected to reduced detoxification rate of GSTP1 substrates and subsequent slower removal of cytotoxic agents from the cells.

Gastrointestinal toxicity of FAC chemotherapy in our study had several predictors belonging to the metabolic pathways of FAC drugs. One of them was the *CBR1* gene, encoding the anthracycline metabolizing enzyme carbonyl reductase 1. Its silent polymorphism p.Ala209= (rs20572) elevated the risk of recurrent vomiting in our analysis. This enzyme is a predominant human doxorubicin reductase in the liver. It is suspected then, that variability in *CBR1* expression may be reflected in interindividual differences in outcome and tolerance of anthracycline therapy [73]. It has been experimentally proved that the polymorphic variants in *CBR1* gene can alter the doxorubicin pharmacokinetics and drug disposition, and also predict anthracycline-related toxicity, but the results are conflicting. Polymorphism p.Ala209= has been shown to increase doxorubicin clearance, decrease its exposure and also peak plasma concentration of doxorubicinol [74]. Conversely, the group of Jordheim observed significant correlation between this SNP and occurrence of grade 3-4 toxicities and treatment delay in patients with diffuse large B-cell lymphoma treated with doxorubicin-containing regimes [75].

The presence of rare allele of polymorphism p.Pro329Ala (rs2228100) in component of cyclophosphamide metabolic pathway, aldehyde dehydrogenase *ALDH3A1* gene, influenced the risk of recurrent anemia and leukopenia. p.Pro329Ala (rs2228100) has been originally detected in patients with Sjögren-Larsson Syndrome, but was described as common and benign [76]. The group of Afsar reported, that the variant allele was predominant in the breast cancer patients treated with FAC chemotherapy [7]. The same group later showed, that wild type homozygote had higher clearance of cyclophosphamide when compared to other genotypes. Suggested explanation was based upon the possibly increased *ALDH3A1* expression, but the data regarding such connection are contradictory [77]. The link between *ALDH3A1* expression and decreased sensitivity to cyclophosphamide has been presented by Sládek [78], while the group of Ekhart saw no impact of *ALDH3A1*genotypes on the drug pharmacokinetics [57]. Findings of Afsar’s group to some extent support our observations, while it has to be mentioned that the authors did not report any connection of *ALDH3A1* SNP to FAC toxicity. Seen in our group the high risk of myelotoxicity symptoms in the presence of p.Pro329Ala genotypes containing rare G allele could be the result of slower detoxification rate of cyclophosphamide and subsequent drug accumulation in the cells, but such statement needs a strong experimental support.

In our group two independent predictors of severe neutropenia, variants in *TYMS* and *DPYD* genes, belong to 5-fluorouracil pathway. It has been reported that patients with low neutrophil count had up to 9 times higher 5-FU levels [79], thus our result confirms the important role of this drug in developing neutropenia. *TYMS* and *DPYD* genes are crucial for 5-FU action, *TYMS* gene encodes the tymidylate synthase, the main cellular target of 5-FU, while the DPYD product, the dihydropyrimidine dehydrogenase, is its key rate-limiting enzyme [9]. In our study the 3R3R genotype of 28bp repeat VNTR variant in *TYMS* gene (rs34743033) was responsible for elevated risk of severe neutropenia. This result is somewhat surprising, as usually the 2R2R genotype is reported as a predictor of 5-FU toxicity [80–82]. The 3R allele has 3-4-fold higher translational efficiency when compared to 2R and the 2R-related TYMS underexpression and enhances the risk of toxic events [82, 83]. The source of inconsistency between our results and those reported by others may be the further inner variation within the *TYMS* VNTR. In 2003 Mandola and coworkers described polymorphism G/C in the second 28bp repeat of 3R allele (rs2853542) which reduces its transcriptional activity to this of 2R allele [84]. Furthermore, later the group of Gusella reported the G/C change in 12th nucleotide in both repeats of 2R allele and assumed, that the 2RC variant would have even lower activity than the 2R itself [85]. Given the data it is possible, that the 3R3R genotype group in our study could be in fact heterogenous. Therefore, the effect on the severe neutropenia risk is more complex and further genotyping of the VNTR internal G/C changes is needed.

There are strong evidences, that systemic low activity of dihydropyrimidine dehydrogenase (DPD) is associated with high risk of severe toxicity of 5-FU, mainly hematological and gastro-intestinal. Patients with partial *DPYD* gene deficiency and subsequent impaired DPD activity were shown to have 3-4-fold higher risk of 5-FU-related neutropenia [9]. Polymorphism p.Ile543Val (rs1801159, DPYD*5) studied in our work has been described as common variation, present at similar frequencies in healthy subjects and in cancer patients [86]. However, there is no conclusion regarding its predicted impact on DPD activity. Offer and colleagues performed functional analysis of several *DPYD* variants and found that p.Ile543Val did not affect enzyme activity [87]. Opposite observations came from the work of Zhang and others, where this polymorphism contributed to lower DPYD activity and toxicity of 5-FU in gastric and colon cancer patients. The variant was responsible for accumulation of 5-FU and treatment-related side effects [88]. In other study the AA homozygote was also overrepresented in the responsive patients [89]. Similarly in the work of Panczyk [90], rs1801159 is described as the expression decreaser. Our results are in opposition to the above-cited studies. High risk of severe neutropenia for the common homozygote AA seen in our group would rather suggest the enhanced *DPYD* activity in presence of p.Ile543Val polymorphic allele. Such inconsistencies may reflect the differences in diagnosis, treatment regimens and ethnicity between studies.

### Polymorphisms in transporter genes

In this study polymorphic variants in genes encoding membrane transporters, both influx (*SLC22A16*) and efflux ones (*ABCB1*, *ABCG2*, *ABCC2*), were the independent predictors of all studied symptoms of FAC myelotoxicity, excluding overall leukopenia. Apart from that, polymorphisms of *SLC22A16* and *ABCC2* were also the factors influencing the gastrointestinal events. These observations emphasize the importance of transport systems to the tolerance of the treatment, as the ones directly involved in maintaining drugs concentration inside the cells. *SLC22A16* was the only influx transporter gene analyzed in this study. This carrier is a known doxorubicin importer, therefore its optimal activity is crucial for the positive treatment outcome. Overexpression is correlated with increasing influx and subsequent growing susceptibility to cytotoxic effects of doxorubicin. Furthermore, because its expression in normal adult tissues seems to be restricted to hematopoietic cells in bone marrow, SLC22A16 transporter must have an important role in hematopoiesis [91]. Also, bone marrow would be especially sensitive to changes in the intake of SLC22A16 substrates. In our study the risk of recurrent neutropenia was elevated for the common homozygote AA of silent polymorphism p.Asn104= (rs6907567). This result is concordant with the findings of Bray *et al*. [10], who observed lower incidence of dose delay, caused by leukopenia and neutropenia, of doxorubicin/cyclophosphamide chemotherapy in breast cancer patients for several SNPs in *SLC22A16*, including p.Asn104=. Another *SLC22A16* variant analyzed in our work, p.His49Arg (rs714368) and the presence of its common allele C was the independent predictor of early nausea. This result is similar to the one obtained by the group of Lal [92]. They described that breast cancer patients harboring the CC genotype had significantly higher exposure levels of doxorubicin and its major metabolite doxorubicinol, when compared to the common homozygote. It is assumed then, that the presence of p.His49Arg G allele could have implications in adverse effects such as nausea and vomiting, which have established correlations with exposure levels to doxorubicin.

The efflux ABC transporters are known for their broad substrate specificity, including cytotoxic drugs. Anthracyclines are, among many others, the substrates for *ABCB1* gene product, the P-glycoprotein. This transmembrane carrier is highly expressed in apical surface of epithelial cells in many locations. Also, its expression in hematopoietic, NK, dendritic cells and in T and B lymphocytes accentuates the *ABCB1* importance for hematopoietic and immune systems [93, 94]. Our results showed that the presence of common homozygote CC of *ABCB1* polymorphism p.Ile1145= (rs1045642) increased the risk of recurrent anemia. Variant p.Ile1145=, albeit the silent one, has been shown to decrease the mRNA stability and transporter activity, probably due to the linkage to the other variant [95, 96]. Despite the known function of p.Ile1145= polymorphism the reports about its influence on chemotherapy outcome and toxicity are not consistent. Observations similar to ours were made by the group of Tsai [97], where the breast cancer patients, the carriers of CC genotype, tended to develop leukopenia after administration of docetaxel. Also, the study of Tatakuwa *et al*. [98] demonstrated that the carriers of wild type genotype were at high risk of neutropenia among lung cancer patients treated with anthracycline amrubicin. These findings are somewhat surprising in the light of the results of the experiments with transduction of *ABCB1* gene into murine hematopoietic cells, where the protection from toxicity of chemotherapeutic agents and increased tolerance to treatment were observed [99]. It could then be assumed that the opposite situation, i.e. with lower *ABCB1* activity, will result in higher sensitivity to drugs. In accordance with these experimental data, but in opposition to the above cited and our findings, are the results of Ikeda and others [100] from the study on breast cancer patients receiving doxorubicin and cyclophosphamide. They observed no correlation between p.Ile1145= and severe neutropenia, although the proportion of symptom cases tended to be higher in TT carriers. Similarly Kim *et al*. [101] presented that breast cancer patients being TT homozygotes had increased risk of neutropenia and diarrhea after docetaxel and doxorubicin chemotherapy. Such inconsistencies may reflect the diagnosis, treatment and ethnicity differences, but also the number of ABCB1 substrates in the cells and the delicate interplay between cellular transporters and metabolizers.

In our study polymorphism p.Gln141Lys (rs2231142) in *ABCG2* gene was another high risk factor of FAC-induced anemia. Because ABCG2 is expressed, among other locations, in digestive tract and hematopoietic stem cells, high transporter activity may reduce the risk of hematological and GI toxicity of drugs being ABCG2 substrates. Variant p.Gln141Lys has been proved to significantly lower the protein expression and subsequently enhance the sensitivity to anticancer drugs [102], which is consistent with our observations of high risk of hematological toxicity in the presence of variant allele A. Increased sensitivity to neoadjuvant anthracycline-based chemotherapy for this polymorphism was also reported by the group of Wu [103] in breast cancer patients group. Similar effect was described by Tian *et al*. [104] in ovarian cancer patients receiving platinum and taxane-based chemotherapy.

The *ABCC2* was third ABC efflux transporter gene in our study that changed the risk of hematologic toxicities, also the only exporter connected with gastrointestinal events. ABCC2 transporter is crucial for glutathione, glucuronide and sulfate conjugates of many drugs, thus it mediates their intracellular concentration. Variant p.Val417Ile (rs2273697) has been shown in *in vitro* models to decrease the apparent affinity for substrates conjugated with glutathione and glucuronide, and to decrease the transport activity [105, 106]. Opposite findings came from the work of Haenisch and colleagues on bioavailability of talinolol, who reported that p.Val417Ile variant was associated with higher activity of intestinal ABCC2 [107]. Taken under consideration, that in our group high risk of overall anemia was seen for the common GG homozygote, our results seem to support the observations made by Haenisch. Wild type homozygote, when compared to overactive Ile417-related ABCC2 transporter, must result in lower activity, which could be the reason of toxic drug accumulation. The second *ABCC2* polymorphism in our study that influenced severe neutropenia and early nausea risk was the silent change p.Ile1324= (rs3740066). Unfortunately, literature does not explain the specific linkages of *ABCC2* p.Ile1324= (rs3740066) 3972C>T polymorphism with the occurrence of over-reactivity to FAC chemotherapy. Functional analysis on the human liver samples revealed that T allele correlated with higher *ABCC2* mRNA expression levels [108]. Despite the silent character, p.Ile1324= variant and its rare homozygote TT is a significant risk factor for intrahepatic cholestasis of pregnancy [109]. The authors hypothesized that this SNP could be detrimental to gene function by, for instance, alternative splicing regulation by disrupting exonic splicing enhancer or silencer-binding elements. Also, this polymorphism had been shown to increase the AUC of irinotecan and its metabolites in patients with solid tumors, and to be the factor of drug resistance in epileptic patients [110, 111].

### Impact of patients clinical characteristics on FAC toxicity

The study revealed that patients’ age was one of the important predictors of tolerance to treatment. Interestingly, both pre- and post-menopausal age changed the risk of different kinds of chemotherapy toxicities. Among nephrotoxicity factors established in this study the patients’ postmenopausal age was the strongest one. This result stays in accordance with general observations of higher proportion of nephrotoxicity in older patients. They usually have reduced total body mass and changed body composition, as well as decreased renal function [112]. The aging kidneys’ susceptibility to nephrotoxicity come as well from chronic inflammation, oxidative stress and cells’ senescence, which lead to hypofiltration, altered renal vasculature and tubule-interstitial changes [20]. The kidneys’ ageing is also reflected in genetic material of kidney cells. Melk *et al*. performed cDNA profiling of human kidney and discovered over 500 genes differently expressed with ageing. Older organs overexpressed proteins of the immune response and inflammation, had increased extracellular matrix synthesis and turnover. On the other hand the glucose and lipid metabolism as well as mitochondrial function were significantly reduced [113].

Premenopausal age of patients was, in turn, the only clinical factor of gastrointestinal toxicity. Young age together with female gender, history of low alcohol intake, experience of emesis during pregnancy, motion sickness and previous contact with cytotoxic drugs are established risk factors of nausea and vomiting after chemotherapy [114]. The reason of age-related differences in response to treatment regimens are difficult to explain. It is well known that pharmacokinetics is changing with age, together with the toxicity of antineoplastic drugs and their metabolites. It can be explained by the fact, that the body with time alters composition, progressively accumulates fat an loses water. The prevalence of nausea and vomiting in younger women in pre-menopausal age seen in our group is consistent with other studies [115]. Also, the clinical observations suggest, that the tendency to GI toxicity in younger cancer patients may be emotionally determined, which is described as anticipatory nausea and vomiting (ANV). Kamen and colleagues associated nausea and vomiting with psychological mechanisms and demographic factors that contributed to the onset, frequency, severity, and duration of ANV. Three distinct but interrelated factors contributing to ANV are: classical conditioning, which may lead to anticipatory nausea; demographic, clinical, and treatment-related factors, which can predict risk to anticipatory nausea; and also the anxiety or negative expectancies [116].

The high risk of overall and early anemia in the subgroup of patients with TNBC seen in our study may be connected to the deficiency in DNA repair system. It has been shown, that triple negative cancers have reduced expression of NER, Fanconi Anemia pathways genes, *RRM1*, *BRCA1* and *CHK1* gene when compared to other types of breast cancers [117, 118]. Because in our group triple negativity was the anemia predictor together with variant in NER’s component *ERCC1*, one could cautiously hypothesize, that the reduced *ERCC1* expression in TNBCs is to some extent reflected in the expression on the patients’ level. Unfortunately, the data regarding the very expression of *ERCC1* in patients with TNBC alone is lacking, but although the tumor genome considerably differs from the host genome, it is plausible that it still carries some resemblance. Nonetheless, our result emphasizes the distinct nature of triple negative breast cancer in terms of chemotherapy tolerance.

### Simultaneous impact of high risk factors on the toxicity of FAC chemotherapy

The results of our cumulative analyses of FAC toxicity risk in presence of independent factors established in this study confirmed multifactorial genesis of adverse reactions. Based on known or suspected functions of polymorphic variants, specific genotypes or clinical features, we attempted to look into the hallmark of modifications needed for developing adverse reactions to treatment. It is crucial to point out, that such interpretations could have only preliminary character and must be treated with caution. Specific and meticulous experimental support is needed for elucidate the background of chemotherapy toxicities.

In our group the elevated creatinine level had three independent predictive factors, which, when present together, were responsible for very high nephrotoxicity risk. Our results showed that the development of this symptom was eased by the active GSTT1 and GSTM1 enzymes, the impairment of DNA repair machinery and by the hallmark of changes occurring during ageing. The strength of our model is emphasized by the lack of nephrotoxicity cases in the subgroup of non-carriers. It is therefore expected, that the actual symptom risk for the presence of all three factors is even higher, since – for mathematical reasons - the reference group included the carriers of one factor. It is also crucial to point out the fact that in our group the older age and active GST enzymes were the nephrotoxicity predictors. The mutually enhanced influence in cumulative analysis is supported by the observations done by Hashimoto and colleagues. They proved in an animal model, that GSH level in liver and kidneys seems to be age-dependently decreased, and was accompanied by the changes in blood chemical parameters, including levels of aminotransferases, creatinine, urea nitrogen and others [119]. Furthermore, the third predictor, the *ERCC1* low expression variant completed the picture of factors interdependence, as the ERCC1 is not only engaged in NER pathway, but also in removal of reactive oxygen species [120]. For the carriers of three independent predictors of nephrotoxicity the supposed sequence of events would be as follows: the impact of doxorubicin, older age and active GST enzymes result in the lowering of cellular GSH level reflected by oxidative damage to the cells, the repair capacity of which is suppressed by the impaired ERCC1-related machinery.

Overall anemia (grade 1–2) high risk genetic factors belonged to all three functional groups – DNA repair (*ERCC1*, p.Asn118=), metabolism (*GSTT1* and *GSTM1* present) and transport (*ABCC2*, p.Val417Ile), with addition of breast cancer triple negativity. The risk of anemia for carriers of all those four factors (i.e. the 4’s) was in the borderline significance (*p* = 0.055), but this could be the result of small number of such cases (*n* = 4). Nonetheless, we hypothesized that the high toxicity risk for the 4’s could be an effect of DNA damage accumulation, hematopoiesis disturbances, slow drugs efflux, GSH depletion and consequent oxidative stress. This hypothesis and relevance of our model is further emphasized by lack of anemia cases in the group of non-carriers (0’s). Similar protective effect in the 0’s group was observed in the analysis of recurrent anemia where the risk of anemia seen in at least 4 cycles of treatment for the carriers of two independent risk factors could arise from slower detoxification rate and drugs accumulation (*ALDH3A1*, p.Pro329Ala) and from simultaneous alterations in efflux by the multidrug transporter (*ABCB1*, p.Ile1145=). Modifications in drugs export and activity of metabolic pathways seems to influence also early anemia symptoms. The presence of all three high risk genotypes was crucial for developing severe neutropenia. Those factors were responsible for alterations in efflux system (*ABCC2*, p.Ile1324=), lower 5-FU detoxification rate with subsequent drug accumulation (*DPYD*, p.Ile543Val) and also for changes in the 5-FU cellular target itself (*TYMS*, 28bp tandem repeat). From the other hand, altered doxorubicin intake by the *SLC22A16* variant (p.Asn104=) together with impaired drugs elimination (*CYP2C19* p.Pro227=) and slower DNA damage repair with hematopoiesis disturbances (*ERCC1* c.1510C>A) seemed to be the basis of recurrent neutropenia. In comparison to above described models, the background of leukopenia in our study differs from other toxic symptoms, because the two high risk factors belonged only to the metabolic group. It is plausible that strongly significant (*p* = 0,0005) risk for the 2’s is the effect of accumulation of cytotoxic agents driven by weakened detoxification rate (*ALDH3A1*, p.Pro329Ala and *CYP2C19*, c.-806C>A).

Both severe early gastrointestinal toxicities, nausea and vomiting, were dependent on the combination of young age of breast cancer patients and modifications in DNA repair machinery (*XRCC1*, p.Arg399Glu and *ATM*, p.Asp1853Asn). It is possible, that emotional impact on GI toxicity, which is often clinically observed in younger women, has some psychophysical effect, that is complemented by the accumulation of DNA damage and changes in rate of intestinal epithelial renewal. An interesting result came from the cumulative analysis of early nausea, as it was the only one where simultaneous changes in transport systems alone (ABCC2, p.Ile1324= and *SLC22A16*, p.His49Arg) were responsible for strongly significant risk elevation. Furthermore, the lack of early nausea cases in the 0’s group emphasize the importance of transport activity for developing this symptom. It could be cautiously hypothesized, that for early GI toxicity the essential point is to overstep some concentration threshold of cytotoxic drugs by changing their import and export, before even the detoxification and other metabolic pathways start off. Finally, carriers of combinations of modified genes of DNA repair and drugs’ metabolic pathways were at high risk of overall nausea and recurrent vomiting. In case of nausea, three independent predictive factors seemed to influence detoxification processes (*CYP1B1*, p.Leu432Val and *GSTP1*, p.Ile105Val) as well as apoptosis/repair balance and thus renewal of intestinal epithelium (*ATM*, p.Asp1853Asn). Similar mechanism was seen for recurrent vomiting, where the high risk applied to carriers of at least one genetic modification – one that may alter doxorubicin reduction (*CBR1*, p.Ala209=) or one in gene belonging to the DNA repair machinery (*TP53* p.Arg72Pro). In our group there were no carriers of both of those high risk factors in analysis of recurrent vomiting.

In conclusion, we found the strongest associations between concurrent presence of clinical factors - overall and recurrent anemia, nephrotoxicity and early nausea and genetic polymorphisms in genes responsible for DNA repair, drugs metabolism and transport pathways. Furthermore, in the cumulative analyses of these adverse effects, the absence of high risk factors was reflected by the lack of given symptom. These results indicate the possibility of selection of the patients with expected high tolerance to FAC treatment and consequently with high chance of chemotherapy completion without the treatment delays and decline in the quality of life.

Our results point out that the complex nature of adverse effects during FAC breast cancer therapy is the interplay among the polygenic inheritance and clinical risk factors. Similar models that engage multiple factors could be potentially useful in future personalized approach to cancer treatment. Currently, the patients who share the same diagnosis are usually treated with the same standard chemotherapy regimens, and any modifications can be made only after adverse reaction occurs. The tool that enable the separation of patients group in terms of expected toxicity and therefore allows the tailoring of treatment to the characteristics of given patients, could significantly improve its tolerance, patients’ quality of life and also outcome. Current clinical protocols for FAC regimen do not foresee the reduction of dose of any of FAC components. However, there are several ways of additional pretreatment that could be implemented. The patients endangered by chemotherapy-induced anemia and by the decrease of white blood cells count (i.e. leukopenia and neutropenia) can be supplemented with iron, folic acid and B6 vitamin. Although the use of erythropoiesis-stimulating agents and colony-stimulating factors is not advised prior to myelotoxic events, the genotype-based selection of high-risk patients could be the expletive indication to those therapies. For the patients with predicted nephrotoxicity the advice of a nephrologist could be recommended, as well as the rehydration. The modifications in the antiemetic treatment, together with special diet, would be needed for the women with genetic markers of gastrointestinal toxicity. Moreover, the young patients from this group are likely to benefit from the psychological consultation, due to the ANV phenomenon and emotional component of GI toxicity.

## MATERIALS AND METHODS

### Patients and samples

A total of 324 women from the Silesian voivodeship, diagnosed with breast cancer were recruited to this study, with the exception of DCIS, LCIS, and Paget tumor cases. The group had been checked for the status of the Silesian-most -common germline mutations in *BRCA1* and *BRCA2* genes (c.68_69delAG, c.181T>G, c.4034delA, c.5266dupC in *BRCA1* and c.5946delT, c.9403delC in *BRCA2* gene). The chosen study group was composed only from non-carriers.

The diagnoses were done between 1997 and 2012, but majority of patients (289; 89.2%) were diagnosed between 2005 and 2009. For all the women the first-line chemotherapy regime was FAC which combines doxorubicin (50 mg/m^2^), 5-fluorouracil (500 mg/m^2^) and cyclophosphamide (500 mg/m^2^). At the Cancer Center in Gliwice the patients who were diagnosed with cardiovascular disease or with low left ventricular ejection fraction did not qualify for FAC treatment. The drugs were administered intravenously on the first day of a 21-day cycle (six planned cycles). The chemotherapy was given in adjuvant or neoadjuvant setting. Baseline blood test results obtained directly before the start of chemotherapy showed that the studied group was free of preexisting adverse symptoms.

All patients filled the informed consent form and agreed to have their samples used for research purposes. This study was approved by the appropriate bioethical committee. Full characteristics of the group under study is given in Supplementary Table 2. The observation ended on the 1st of March 2015.

### Polymorphisms selection

For this study we selected genes (and their polymorphic variants) belonging to three major functional groups. First, we chose the variants with known or potential role in transport and metabolism pathways of all three FAC drugs. Second group consisted of genes being part of DNA damage repair systems because of the destructive impact of chemotherapy drugs on cellular DNA. The last group contained intracellular FAC drug targets. The data for variants selection were extracted from the Pharmacogenomics Knowledge Base (www.pharmgkb.org) and the NCBI databases: dbSNP (www.ncbi.nlm.nih.gov/snp) and PubMed (www.ncbi.nlm.nih.gov/pubmed/).

### Genotyping

Genomic DNA was isolated from the peripheral blood leukocytes using the phenol-chloroform method or commercial DNA isolation kits. Genotyping was performed using RFLP-PCR, multiplex-PCR or allele-specific amplification PCR (ASA-PCR) methods. PCR reagents purchased from Applied Biosystems (AmpliTaq Gold Polymerase) and EURx (Perpetual Taq Polymerase) were used. Genotyping of polymorphic variants in *ABCB1* (rs1045642, rs2032582, rs1128503), *ABCC2* (rs2273697, rs717620, rs3740066), *ABCG2* (rs2231142),*SLC22A16* (rs714368, rs12210538) *MTHFR* (rs1801133), *DPYD* (rs1801159), *GSTP1* (rs1695), *CYP1B1* (rs1056836), *CYP2C19* (rs4244285), *TYMS* (rs34743033), *ERCC1* (rs11615, rs3212986), *ERCC2* (rs13181), *XRCC1* (rs25487), *TP53* (rs1042522), *ATM*(rs1801516) as well as detection of *GSTT1*/*M1* deletions were performed as described previously [27]. The genotyping methods for polymorphisms in *ABCB1* (rs1128503), *SLC22A16* (rs6907567, rs723685), *ALDH3A1* (rs2228100), *CBR1* (rs20572) and*CYP2C19* (rs12248560) were developed for this study. Primers were designed with Primer3 web application (http://primer3plus.com/) or extracted from the literature. RFLP methods including restriction sites implementation were designed using the WatCut online tool (http://watcut.uwaterloo.ca/). Primer sequences and expected amplification products were verified using NCBI BLAST tools (http://blast.ncbi.nlm.nih.gov/Blast.cgi) and *in silico* PCR (http://genome.ucsc.edu/cgi-bin/hgPcr). PCR or digestion products were separated on agarose gels stained with ethidium bromide. For the quality control randomly selected samples of each genotype were checked by direct sequencing with full result accordance. Genotyping methods are summarized in Table 2.

**Table 2:** Genotyping methods

Category	Gene	Ref SNP ID	Alleles wt/v	Mutation	Method	Enzyme	Primer sequences 5′→3′	Method source
transporters	*ABCB1*	rs1045642 rs2032582 rs1128503	C/T G/T/A C/T	p.Ile1145= p.Ser893Ala/Thr p.Gly412=	RFLP RFLP RFLP RFLP	MboI RsaI (vA) BbvI (vT) HaeIII	F: ttgatggcaaagaaataaagc; R: cttacattaggcagtgactcg Fcommon: agcaaatcttgggacaggaa; RA: agtccaagaactggctttgc Fcommon: agcaaatcttgggacaggaa; RT: tat ttagtttgactcaccttc**G**ca F: tgacagctattcgaagagtg; R: aagggcaacatcagaaagat	[27] [27] own
	*ABCC2*	rs2273697 rs717620 rs3740066	G/A C/T A/G	p.Val417Ile c.-24C>T; 5’-UTR p.Ile1324=	RFLP RFLP RFLP	NcoI BbsI SfaNI	F: ggcaaagaagtgtgtggat; R: acatcaggttcactgtttctcc**C**a F: taaatggttgggatgaaagg; R: gctttagaccaattgcacatc F: tggctgctatccttccctct; R: ctcagagggatcacttgtg**G**ca	[27] [27] [27]
	*ABCG2*	rs2231142	C/A	p.Gln141Lys	ASA PCR	--	F: tagcaggctttgcagaca t; R: caagccacttttctcattgtt RC: gaagagctgctgagaactgtaag; RA: cgaagagctgctgagaactt	[27]
	*SLC22A16*	rs714368 rs12210538 rs6907567 rs723685	A/G A/G A/G T/C	p.His49Arg p.Met409Thr p.Asn104= p.Val252Ala	RFLP RFLP RFLP RFLP	FokI StyI FaqI AluI	F: tggagacccttcaaatttgct; R: gggcctgcagaca**G**ga F:ccaggttaggcttttctttt; R:ttgctcaatgacaggtgtag F: ctggatcagcattgcaagcc; R: gctctcaaggtgtagcagg**G** F: gtggggtttgtctatgtgat; R: agtgtccttttcgtaatgct	[27] [27] own own
drugs metabolizers	*ALDH3A1*	rs2228100	C/G	p.Pro329Ala	RFLP	MspI	F: gggtctaggtgcttgcactt; R: gcctccatctcctgctcttc	own
	*CBR1*	rs20572	C/T	p.Ala209=	RFLP	BfaI	F: gtggtgaacgtatctagcat; R: accactgttcaactctcttc	own
	*DPYD*	rs1801159	A/G	p.Ile543Val	RFLP	PsiI	F:ttttgcagtcacaatatgga; R:tcaaaagctcttcgaatcat	[27]
	*MTHFR*	rs1801133	C/T	p.Ala222Val	RFLP	TaqI	F: tgaaggagaaggtgtctgcggga; R: aggacggtgcggtgagagtg	[27]
	*GSTT1*	--	+/−	gene deletion	multiplex PCR	--	T1-F: tctccttactggtcctcacatctc; T1-R: tcaccggatcatggccagca M1-F: gaactccctgaaaagctaaagc; M1-R: gttgggctcaaatatacggtg β-globin-F: gaagagccaaggacaggtac; β-globin-R: caacttcatccacgttcacc	[27]
	*GSTM1*	--	+/−	gene deletion				
	*GSTP1*	rs1695	A/G	p.Ile105Val	RFLP	Alw26I	F: accccagggctctatgggaa; R: tgagggcacaagaagcccct	[27]
	*CYP1B1*	rs1056836	C/G	p.Leu432Val	RFLP	AcuI	F: gcctgtcactattcctcatgcc; R: gtgagccaggatggagatgaag	[27]
	*CYP2C19*	rs4244285 rs12248560	G/A C/T	p.Pro227= c.-806C>A	RFLP RFLP	MspI SfaNI	F: aattacaaccagagcttggc; R: tatcactttccataaaagcaag F: cccttagcaccaaattctct; R: atttgagctgaggtcttctg	[27] own
5-FU target	*TYMS*	rs34743033	2R/3R	28bp tandem repeat	PCR	--	F: gtggctcctgcgtttccccc; R: gctccgagccggccacaggca	[27]
DNA repair	*ATM*	rs1801516	G/A	p.Asp1853Asn	RFLP	Sau3AI	F: taatatgtcaacggggcatg; R: atttctccatgattcatttg**G**at	[27]
	*ERCC1*	rs11615 rs3212986	T/C C/A	p.Asn118= c.1510C>A	RFLP RFLP	BsrDI MboII	F: aggaccacaggacacgcaga; R: catagaacagtccagaacac F: cagagacagtgccccaagag; R: gggcaccttcagctttcttt	[27] [27]
	*ERCC2*	rs13181	T/G	p.Lys751Gln	RFLP	PstI	F: ccccctctccctttcctctgttc; R: ggacctgagcccccactaacg	[27]
	*XRCC1*	rs25487	G/A	p.Arg399Glu	RFLP	MspI	F: ttgtgctttctctgtgtcca; R: tcctccagccttttctgata	[27]
	*TP53*	rs1042522	G/C	p.Arg72Pro	RFLP	Bsh1236I	F: tcccccttgccgtcccaa; R: cgtgcaagtcacagactt	[27]

bolded bases in capital letters indicate introduction of restriction site; SNP- single nucleotide polymorphism; wt- wild type; v- variant.

### Study endpoints

The aim of the present study was to investigate the influences of polymorphic variants in genes related to FAC drugs on the treatment-induced toxicity in search for risk factors. All the adverse events that occurred at any point of the first-line treatment were evaluated according to EGOG *Common Toxicity Criteria*. The hematological toxicities included anemia, neutropenia, thrombocytopenia and leukopenia. Nausea and vomiting belonged to gastro-intestinal toxicity, whereas liver and kidneys damage belonged to non-hematological group of symptoms. All the events were categorized by the time of occurrence (overall - during whole first course of chemotherapy, and early – during the first two cycles) and the severity of symptoms (toxicity of any grade, and severe - grades 3 and 4) (Table 3). Apart from standard ECOG grading we also analyzed the duration of a given symptom during the first course of treatment. The symptom was defined as recurrent if it was present during four or more cycles (for any grade). Recurrent severe events (grades 3 and/or 4) were seen during at least two cycles. In our group recurrent anemia of any grade was present in 9 (9.2%), leukopenia in 38 (11.7%), nausea in 42 (13.0%) and vomiting in 6 (1.9%) of patients. Recurrent severe neutropenia afflicted 21 (6.5%) women, nausea was seen in 10 (3.1%) and vomiting in 3 (0.9%) of patients. Other symptoms had not been present in the recurrent mode.

**Table 3:** Frequency of FAC chemotherapy toxicities by the ECOG grade

Category	Symptom	Toxicity grade. ECOG *Common Toxicity Criterian* (%)					Number of valid cases
		**0**	**1**	**2**	**3**	**4**	
OVERALL TOXICITY during the whole first course of treatment	Anemia	291 (90.1)	20 (6.2)	12 (3.7)	–	–	323
	Neutropenia	148 (45.7)	21 (6.5)	93 (28.7)	54 (16.7)	8 (2.4)	324
	Thrombocytopenia	319 (98.5)	5 (1.5)	–	–	–	324
	Leukopenia	146 (45.1)	127 (39.2)	47 (14.5)	4 (1.2)	–	324
	Hepatotoxicity	215 (69.4)	88 (28.4)	6 (1.9)	1 (0.3)	–	310
	GI toxicity - nausea	122 (37.7)	78 (24.1)	85 (26.2)	39 (12.0	–	324
	GI toxicity - vomiting	238 (73.5)	24 (7.4)	44 (13.6)	18 (5.5)	–	324
	Nephrotoxicity	292 (95.4)	11 (3.6)	3 (1.0)	–	–	306
EARLY TOXICITY during first two cycles of treatment	Anemia	305 (94.7)	9 (2.8)	8 (2.5)	–	–	322
	Neutropenia	205 (63.7)	9 (2.8)	70 (21.7)	33 (10.2)	5 (1.6)	322
	Thrombocytopenia	319 (99.1)	3 (0.9)	–	–	–	322
	Leukopenia	208 (64.4)	87 (26.9)	27 (8.4)	1 (0.3)	–	323
	Hepatotoxicity	266 (88.4)	33 (11.0)	2 (0.6)	–	–	301
	GI toxicity - nausea	172 (53.2)	69 (21.4)	61 (18.9)	21 (6.5)	–	323
	GI toxicity - vomiting	260 (80.2)	27 (8.4)	25 (7.7)	12 (3.7)	–	324
	Nephrotoxicity	287 (97.3)	5 (1.7)	3 (1.0)	–	–	295

As an addition to potential genetic determinants of FAC toxicity we performed similar analyses for the set of clinical factors including TNM staging, receptors status, TNBC, tumor histotype, patient’s age at the time of diagnosis, neo/adjuvant setting of chemotherapy and number of cycles. Factors like radiotherapy and its dosage, immunotherapy, hormonotherapy and follow-up events (death, progression, recurrence, metachronic breast cancer) were not included to our analyses. All these factors occurred after the chemotherapy completion and therefore could not have any impact on immediate FAC toxicity.

### Statistics

The difference between observed and expected genotype frequencies were tested for Hardy-Weinberg Equilibrium (HWE) with the χ^2^ test. Correlations between clinical factors, treatment-related toxicity and polymorphic variants were established with Pearson χ^2^ and Fischer two-way exact tests. A dominant, recessive and co-dominant genetic models were used in all analyses for all the genetic variants. *P <* 0.05 was considered as statistically significant, while *p <* 0.100 was treated as indicator for trend in given analysis.

Genetic and clinical factors that correlated with toxic symptoms in univariate analyses with p-value below 0.100 were included in multivariate analyses. This level was used to include in the model polymorphic variants with possible, but weak, individual impact on the adverse reaction to treatment. The final model of independent predictive factors (*p <* 0.05) of FAC chemotherapy toxicities was established after stepwise regression. Cumulative analyses were performed for the risk of given toxic symptom connected with the concurrent presence of one and more independent predictive factors. Risk analyses were performed using logistic regression model where odds ratios (ORs), 95% confidence intervals (95% CIs) and p-values were calculated. All statistical calculations were performed using Statistica v.10.0 software (StatSoft).

## SUPPLEMENTARY MATERIALS TABLES







## Abbreviations

5-FU: 5-fluorouracil
ALAT: alanine transaminase
ALP: alkaline phosphatase
ANV: anticipatory nausea and vomiting
AspAT: aspartate transaminase
BER: base excision repair
CI: confidence interval
CINV: chemotherapy-induced nausea and vomiting
DCIS: ductal carcinoma *in situ*
ECOG: Eastern Cooperative Oncology Group
FAC: doxorubicin, 5-fluorouracil
GI: gastrointestinal
HER2: human epidermal growth factor receptor-2
ICL: interstrand DNA crosslinks
LCIS: lobular carcinoma *in situ*
NER: nucleotide excision repair
OR: odds ratio
OS: overall survival
PCR: polymerase chain reaction
PFS: progression-free survival
RDW: red (cell) distribution width
RFLP: restriction fragment length polymorphism
RFS: recurrence-free survival
ROS: reactive oxygen species
SNP: single nucleotide polymorphism
TNBC: triple negative breast cancer.
