# Supplementary material for: Pharmacogenetics of toxicity of 5-fluorouracil, doxorubicin and cyclophosphamide chemotherapy in breast cancer patients
Source: Oncotarget. 2018 Jan 10;9(10):9114–36. doi: 10.18632/oncotarget.24148 (PMC5823653; doi:10.18632/oncotarget.24148)
Supplement: Supplementary file 1 [file oncotarget-09-9114-s001.pdf]

Pharmacogenetics of toxicity of 5-fluorouracil, doxorubicin and cyclophosphamide chemotherapy in breast cancer patients

SUPPLEMENTARY MATERIALS

Supplementary Table 1: Multivariate analysis of the associations between SNPs and risk of FAC toxicity. See Supplementary_Table 1

Supplementary Table 2: Characteristics of breast cancer patients group. See Supplementary_Table 2
